# Motor Function in the Setting of Nerve Allografts: Is This the Future of Facial Nerve Reconstruction?

**DOI:** 10.3390/jcm14155510

**Published:** 2025-08-05

**Authors:** Léna G. Dietrich, Adriaan O. Grobbelaar, Ioana Lese

**Affiliations:** Department of Plastic and Hand Surgery, Inselspital, University Hospital, University of Bern, 3012 Bern, Switzerland

**Keywords:** facial nerve reconstruction, peripheral nerve reconstruction, autograft, processed nerve allograft, surgical outcomes, motor recovery

## Abstract

**Background**: Peripheral nerve injuries, especially involving the facial nerve, present unique reconstructive challenges due to their complex functional demands and limited regenerative potential. While autografts remain the gold standard, their drawbacks—such as donor-site morbidity and limited availability—have driven interest in processed nerve allografts. Acellular grafts, in particular, offer promising off-the-shelf alternatives without the need for immunosuppression. **Methods**: We conducted a narrative review of the literature (1990–2023), identifying 55 peer-reviewed studies via PubMed, Embase, and Cochrane Library. The studies included clinical and preclinical work on motor nerve regeneration using processed nerve allografts, with particular attention to outcomes in facial nerve repair. Two independent reviewers conducted abstract screening, full-text review, and data extraction. **Results**: Processed nerve allografts show encouraging motor recovery in gaps under 50 mm, with recovery rates of up to 85% reported. Outcomes decrease significantly in longer gaps (>50–60 mm) and in complex cases, including facial nerve repairs, where evidence remains sparse and largely extrapolated from broader motor nerve data. Registry data (e.g., RANGER) support their use but are limited by heterogeneity and lack of randomization. **Conclusions**: Processed nerve allografts represent a viable alternative to autografts in selected cases—especially short to mid-length motor nerve defects. However, their role in facial nerve reconstruction remains insufficiently studied. Further trials are needed to address specific anatomical and functional challenges in this subgroup and to clarify long-gap efficacy.

## 1. Introduction

Peripheral nerve injuries, particularly facial nerve lesions, significantly impair motor function and severely diminish patient quality of life [1]. Among these, facial nerve injuries present distinct challenges due to their intricate branching pattern, involvement in both voluntary and emotional expression, and the high functional and aesthetic demands of reanimation. Unlike other motor nerves, even subtle deficits can result in social impairment and decreased quality of life. Effective nerve regeneration remains a major clinical challenge due to complex anatomy, limited intrinsic regenerative capacity, and the necessity for precise motor recovery [1,2]. The current clinical guidelines advocate tension-free direct sutures for short nerve gaps (<1 cm) and nerve grafts or nerve transfers for larger defects [1,2]. Autologous nerve grafts, predominantly harvested from sensory nerves such as the sural nerve, are currently considered the gold standard due to their immunological compatibility and inherent regenerative capacity [2,3]. However, autograft procedures entail significant limitations, including donor-site morbidity, sensory loss, and limited availability of suitable donor nerves [2,3].

Processed nerve allografts, particularly acellular nerve grafts such as the Avance^®^ Nerve Graft, have emerged as promising alternatives to autografts [4,5]. The development of these processed allografts overcomes traditional drawbacks such as immunological rejection and the requirement for systemic immunosuppression [4,5,6]. By providing an acellular biological scaffold, these grafts support axonal regeneration and potentially reduce operative morbidity and complications associated with autologous graft harvesting [6,7].

Despite encouraging clinical and experimental outcomes, the comparative efficacy of nerve allografts versus autografts, particularly concerning motor nerve recovery, remains debated due to inconsistent evidence regarding motor function recovery, especially in long-gap reconstructions [8,9,10]. Moreover, although motor nerve regeneration has been widely studied in general, the specific application of processed allografts to facial nerve reconstruction remains underexplored and insufficiently documented. Available clinical data are limited, often anecdotal, and rarely involve controlled or prospective design. This narrative review synthesizes clinical, experimental, and review-based evidence on motor nerve regeneration using processed nerve allografts, with particular emphasis on facial nerve repair. It aims to clarify the current scope of evidence, identify knowledge gaps, and outline the challenges unique to facial nerve reconstruction [9,10,11,12,13].

## 2. Materials and Methods

This work constitutes a narrative review of the literature. A structured but non-systematic literature search was conducted using electronic databases, including PubMed, Embase, and the Cochrane Library, to identify relevant peer-reviewed articles published between January 1990 and December 2024. The search utilized predefined keywords and Boolean operators: “Facial nerve reconstruction”, “peripheral nerve reconstruction”, “autograft”, “allograft”, “surgical outcomes”, and “motor recovery”.

Two reviewers independently screened the identified titles and abstracts based on predefined inclusion and exclusion criteria. The inclusion criteria were peer-reviewed original research articles, case series, systematic reviews, and meta-analyses explicitly reporting on nerve allograft outcomes for motor nerve regeneration. The exclusion criteria involved studies exclusively addressing sensory nerve reconstruction, studies not involving processed nerve allografts, and publications in languages other than English or German.

Articles meeting the initial inclusion criteria underwent full-text review by the same two reviewers independently. Discrepancies regarding article inclusion were resolved through discussion and consensus. Subsequently, data extraction was performed, capturing specific variables including the type of nerve graft (autograft or allograft), nerve defect length and diameter, surgical techniques employed, clinical outcomes in motor recovery (quantified via standardized motor grading systems such as Medical Research Council grading), complications encountered, and the necessity for immunosuppression.

Animal studies that provided comparative preclinical data on nerve regeneration techniques were additionally reviewed and included to complement clinical evidence.

From an initial pool of identified studies, 55 articles ultimately met the criteria for detailed review and data extraction, providing comprehensive insights into clinical outcomes, comparative effectiveness, and the limitations associated with nerve allografts, particularly the Avance^®^ Nerve Graft, in motor nerve reconstruction.

While this review does not follow the PRISMA guidelines and was not prospectively registered, it applies transparent and reproducible selection methods to provide a comprehensive synthesis of the current evidence. The focus lies on the clinical applicability and limitations of processed nerve allografts in motor nerve reconstruction, especially involving the facial nerve.

## 3. Results

A total of 55 publications were included in the present analysis, spanning the period from 1991 to 2024, with the majority published between 2010 and 2023. The included literature comprised 25 clinical studies, 14 experimental animal investigations, and 16 systematic reviews or meta-analyses. Notably, several studies contributed data to more than one category. Among the 55 included studies, only a small subset explicitly addressed facial nerve repair using processed or acellular nerve allografts. Specifically, four preclinical animal studies and one clinical study directly reported facial nerve-specific outcomes. Two additional clinical series included facial nerve cases, but did not analyze them separately. This highlights the current lack of focused clinical evidence for facial nerve allografts.

### 3.1. Clinical Studies

**Figure 1:**
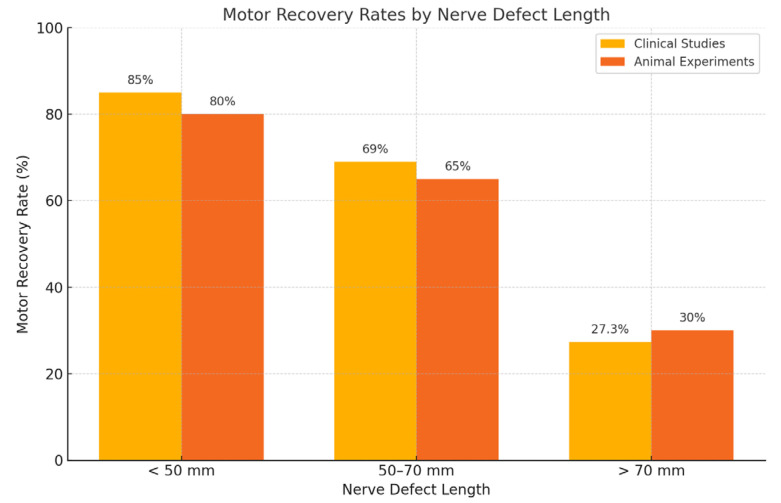
Motor recovery rates by nerve gap length in clinical and preclinical studies. Comparison of motor recovery rates according to nerve defect length in clinical studies and animal experiments. Data show higher success rates in defects < 50 mm, with declining outcomes in longer gaps, particularly those exceeding 70 mm.

**Figure 2:**
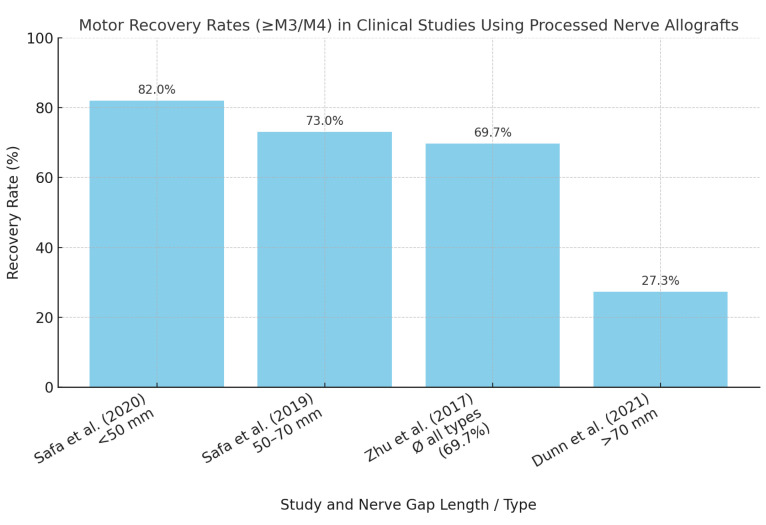
Motor recovery rates in clinical studies using processed nerve allografts. Reported motor recovery rates (defined as ≥M3/M4 according to the MRC scale) in clinical studies evaluating processed nerve allografts. Outcomes vary substantially depending on injury type, nerve gap length, and patient selection criteria [16,17,21,27].

Of the included studies, 25 were clinical original articles and case series [1,2,3,7,8,10,11,12,13,14,15,16,17,18,19,20,21,22,23,24,25,26,27,28,29]. Clinical outcomes demonstrated meaningful motor recovery (≥M3/M4 according to the Medical Research Council scale, assessed via standardized scales) in up to 85% of the cases for shorter nerve gaps (<50 mm), as shown notably by Safa et al. (2020) [16,17]. However, motor recovery significantly decreased to approximately 69% for nerve gaps between 50 and 70 mm [16,17]. Data from the multicenter RANGER registry emphasized the impact of injury type on clinical outcomes, with recovery rates varying substantially from 74% in complex injuries to as high as 94% in neuroma resections [15,16,17]. As shown in Figure 1, motor recovery rates are highest for nerve gaps < 50 mm and decline sharply for defects > 70 mm. This trend is consistent across clinical and preclinical studies, underscoring the biological limitations of long-gap nerve regeneration. Several clinical studies have reported variable motor recovery rates using processed nerve allografts, with outcomes ranging from 27.3% to 86%, depending on nerve gap length, injury complexity, and graft diameter. Detailed outcomes of clinical studies evaluating motor recovery with processed nerve allografts are summarized in Table 1 highlighting variability across indications, nerve types, and study populations. Aggregated from nine clinical studies (n = 733 patients), Figure 2 illustrates reported motor recovery rates using processed nerve allografts, ranging from 27.3% to 86%, depending on nerve gap length, injury complexity, and patient selection.

### 3.2. Experimental Animal Studies

Fourteen experimental animal studies were reviewed [4,5,6,30,31,32,33,34,35,36,37,38,39,40]. These studies generally supported the clinical findings, demonstrating that decellularized nerve allografts facilitate effective motor nerve regeneration, although typically at a slower rate compared to autografts [30,36]. In a primate model of facial nerve repair, Hontanilla et al. demonstrated that although cold-preserved allografts treated with FK506 resulted in similar distal axonal counts and muscle activation compared to autografts, the number of regenerated motoneurons in the facial nucleus was substantially lower. These findings suggest that even when clinical function appears recovered, underlying neurophysiological regeneration may be incomplete in allografts following immunosuppression withdrawal [3]. Specifically, Giusti et al. (2016) and Tang et al. (2019) reported similar functional outcomes between decellularized nerve allografts and autografts for nerve defects up to 10–15 mm [30,36]. Strategies aimed at enhancing allograft performance, such as mesenchymal stem cell augmentation and induced angiogenesis, showed improvements but did not consistently surpass the outcomes of autografts [37,38].

### 3.3. Systematic Reviews and Meta-Analyses–Results

Sixteen systematic reviews or meta-analyses were identified [9,41,42,43,44,45,46,47,48,49,50,51,52,53,54,55]. Reviews by Lans et al. (2023) and Deslivia et al. (2015) indicated comparable rates of meaningful motor recovery between autografts (81.6%) and allografts (87.1%) [29,49,50]. It should be noted that Deslivia et al. included primarily lower-evidence studies (levels IV and V), which limits the generalizability of the reported outcomes. Synthetic conduits, however, exhibited significantly lower success rates (approximately 62.2%) [49]. Optimal clinical outcomes were consistently reported for nerve defects under 60 mm, reinforcing that larger defects, especially for motor or mixed nerves, remain better suited for autografts, pending more robust allograft-specific data [29,49,50].

### 3.4. Outcomes in Facial Nerve Reconstruction

Although clinical data are limited, multiple animal studies have explored the regenerative potential of allografts in facial nerve repair. These include models evaluating functional recovery [5,31], donor nerve types [34], and bioengineering enhancements [9].

Carlson et al. reported on two patients with facial nerve palsy who underwent facial reanimation using cadaveric nerve allografts as part of a babysitter procedure; both showed functional recovery of eye sphincter and lip depressors [25]. Similarly, Leckenby et al. included a small subset of facial nerve injuries in a retrospective allograft study, though outcomes were not analyzed separately, reflecting the lack of dedicated subgroup data [14].

Preclinical models provide stronger support for feasibility. In a primate model, Hontanilla et al. demonstrated successful reinnervation of facial muscles after allograft reconstruction with tacrolimus immunosuppression, although motoneuron counts in the facial nucleus were significantly reduced after withdrawal, suggesting subclinical deficits [5]. Hu et al. confirmed functional recovery in a rabbit model using processed allografts, although histological analysis revealed thinner myelin sheaths and less organized nerve architecture compared to autografts [31]. Zhu et al. extended these findings to xenogeneic acellular grafts in rhesus monkeys, reporting functional muscle activation, though long-term regenerative integrity remained inferior to autografts [32].

The type of donor nerve may also influence outcomes. Ali et al. showed in a rat facial nerve model that motor nerve autografts led to significantly better axonal regeneration and functional recovery compared to sensory nerve autografts, suggesting that donor nerve identity, which is typically undefined in commercial allografts, may influence outcomes [34].

Bengur et al. reviewed current bioengineering approaches in facial nerve models, noting that processed allografts alone may be insufficient for complete recovery and require augmentation via stem cells or growth factor strategies [9].

Overall, facial nerve regeneration using allografts is supported by consistent preclinical data and sparse clinical reports. An overview of relevant studies on facial nerve reconstruction using processed or acellular nerve allografts is provided in Table 2. However, outcomes are often extrapolated from non-facial motor nerve studies, and the unique anatomical and psychosocial roles of the facial nerve limit generalizability. Therefore, conclusions must be interpreted with caution, and further clinical trials with defined facial nerve subgroups and standardized outcome measures are needed.

### 3.5. Limitations and Failures

Despite overall favorable outcomes, several studies described graft failures attributed primarily to poor vascularization, postoperative infections, inadequate surgical techniques, and inappropriate patient selection [26,27,28,54]. Notably, large nerve gaps (>70 mm), increased nerve diameters (>5 mm), and extensive traumatic injuries, such as military blast trauma, resulted in substantially lower success rates, averaging around 27.3% [27]. Furthermore, heterogeneity in study designs and the lack of randomization, particularly in large registry datasets, may introduce bias and limit the comparability of reported outcomes.

## 4. Discussion

Processed nerve allografts represent a promising alternative to autologous grafts for motor nerve repair, particularly in short to intermediate gap lengths. However, only a limited number of studies have explicitly addressed facial nerve repair. In most cases, facial nerve data were embedded within broader cohorts and not reported separately. This is especially relevant because the facial nerve’s complex branching pattern and its dual role in voluntary and emotional expression make even minor functional deficits clinically significant. A comparative overview of the clinical indications, advantages, and limitations of autografts versus processed allografts is summarized in Table 3. This distinction is particularly relevant when considering nerve gap length, donor-site morbidity, and the urgency of surgical intervention.

### 4.1. Historical Development of Nerve Allografts

Although significant advancements have been made in reconstructive surgery, peripheral nerve repair has lagged behind, primarily due to inherent biological constraints such as Wallerian degeneration, which severely limits functional recovery [51]. Autologous nerve grafts, predominantly sensory in origin, remain the gold standard but are limited by donor-site morbidity, sensory loss, and restricted availability. To address these issues, alternative strategies such as synthetic conduits and allografts have been developed. Initially, materials like silicone were used, later followed by biocompatible materials such as collagen and polyglycolic acid [52].

These conduits provided structural support but showed reliable results mainly for short nerve gaps (<25 mm) [52].

Processed nerve allografts represent a significant innovation, providing biological scaffolds capable of bridging nerve discontinuities without the morbidity associated with autografts.

Early allografts required systemic immunosuppression due to immunogenic cellular remnants, thus limiting their clinical utility due to adverse effects and graft rejection [4]. The advent of acellular nerve grafts around 2010 eliminated immunogenic components, effectively eliminating the need for immunosuppression and broadening their clinical applicability [4,5,6]. This technological leap marked the beginning of broader acceptance and clinical implementation, exemplified by commercial products like Avance^®^ Nerve Graft and AxoGuard^®^ Nerve Protector.

Early evaluations by Giusti et al. (2012) advised cautious optimism, highlighting that autografts still provided superior outcomes regarding muscle recovery [35]. Nonetheless, subsequent studies reported successful clinical outcomes in motor nerve reconstruction using cryopreserved allografts, particularly in non-immunosuppressed individuals [3]. Notably, the RANGER registry, initiated in 2008, has now documented over 2000 nerve repairs using acellular nerve allografts, showing steady growth in their use for motor nerve reconstruction [15,16,17,18].

### 4.2. Experimental Studies

Animal studies consistently demonstrate functional motor recovery with processed nerve allografts comparable to autografts, albeit generally slower [30]. Preclinical evidence includes successful nerve regeneration in models ranging from rodents to rhesus monkeys, confirming their broad potential application [31,32]. Orientation and source of grafts influenced early regenerative outcomes in some animal studies, but long-term differences were negligible [33].

Research comparing sensory and motor nerve grafts found similar efficacy, raising questions about the distinct regenerative potentials of graft types [34].

Strategies to enhance regeneration have been extensively investigated. Combined use of mesenchymal stem cells and angiogenesis-promoting techniques has improved outcomes, though autografts typically still outperformed allografts in terms of muscle mass and functional metrics [37,38]. Notably, no significant advantages were observed with vascular endothelial growth factor administration, underscoring the complex interplay between angiogenesis and nerve regeneration [30]. The heterogeneity in experimental findings emphasizes ongoing uncertainty regarding optimal techniques for enhancing allograft performance.

### 4.3. Human Studies

Early clinical experiences with nerve allografts were mixed. Initial reports in the 1990s described limited motor recovery, even with immunosuppression [19,20]. However, advancements in processed acellular allografts have markedly improved outcomes. The RANGER registry provides robust real-world evidence demonstrating meaningful motor recovery rates between 69% and 94%, dependent on injury type and nerve gap length [15,16,17]. Safa et al. reported high success rates, especially in nerve gaps <50 mm, while outcomes decreased significantly beyond this length [16,17]. Additionally, diameter was a critical determinant of success, with smaller diameters consistently yielding superior outcomes [23].

Despite these promising findings, allografts exhibit limitations. Zhu et al. (2017) showed meaningful recovery in only 40% of motor nerve cases, emphasizing gap length as a critical predictor of outcome [21]. Studies have also highlighted the complexity of facial nerve reconstruction specifically, where data remain sparse, and outcomes are variable due to the intricate anatomical and functional requirements [25].

### 4.4. Challenges and Evidence for Facial Nerve Repair with Allografts

The facial nerve’s complex anatomical course and its dual role in voluntary motor function and involuntary emotional expression make it uniquely challenging to reconstruct. Outcomes from upper extremity motor nerves cannot be directly extrapolated to the facial nerve due to differences in fascicular organization, target specificity, and functional demands.

Clinical evidence on the use of processed nerve allografts for facial nerve repair remains sparse. Most published data are derived from broader case series involving mixed nerve injuries, in which facial nerve cases are often underrepresented or not analyzed as a distinct subgroup. For example, Carlson et al. reported two cases of facial nerve repair using cadaveric nerve allografts as part of a babysitter procedure; both patients showed functional recovery of the eye sphincter and lip depressors [25]. Similarly, Leckenby et al. included facial nerve injuries in their cohort of peripheral nerve repairs with processed allografts, but did not provide facial nerve-specific outcome data [14]. This lack of focused clinical data represents a major limitation in the current literature.

Preclinical models have provided stronger support for the feasibility of facial nerve reconstruction with allografts. In a primate model, Hontanilla et al. demonstrated successful reinnervation of facial musculature using cold-preserved allografts under tacrolimus immunosuppression. However, following immunosuppression withdrawal, motoneuron counts in the facial nucleus decreased by approximately 70%, despite preserved clinical function, indicating a risk of subclinical deficits [5]. Hu et al. confirmed functional regeneration in a rabbit model using processed nerve grafts, although histological recovery, particularly myelin sheath thickness, remained inferior to that of autografts [31]. Zhu et al. extended these findings to xenogeneic acellular porcine nerve grafts in rhesus monkeys, reporting functional muscle activation but incomplete structural regeneration compared to autografts [32].

The type of donor tissue may also influence outcomes. In a rat facial nerve model, Ali et al. showed that motor nerve autografts led to significantly better axonal regeneration and whisker movement compared to sensory nerve autografts, suggesting that donor nerve identity, typically undefined in commercial allografts, may impact success rates [34].

Additionally, Bengur et al. emphasized in a recent review that processed allografts alone may be insufficient for complete facial nerve recovery. Emerging bioengineering strategies, such as stem cell augmentation, controlled growth factor release, and topographically guided scaffolds, may enhance regenerative potential, particularly in complex or long-gap facial nerve defects [9].

In summary, while animal models demonstrate the feasibility of facial nerve repair using processed allografts, their clinical efficacy remains unestablished. Standardized outcome measures such as the House–Brackmann and Sunnybrook scales are inconsistently applied, further complicating interpretation. High-quality clinical trials with clearly defined facial nerve endpoints are urgently needed. Future research should prioritize hybrid approaches that combine processed allografts with regenerative technologies to meet both the functional and aesthetic demands of facial nerve reconstruction.

### 4.5. Discussion of Systematic Reviews and Meta-Analyses

Meta-analyses consistently demonstrate comparable outcomes between autografts and allografts for shorter nerve gaps (<60 mm), with significantly poorer outcomes associated with synthetic conduits [49,50]. Notably, Lans et al. demonstrated comparable meaningful recovery rates (87.1% for allografts vs. 81.6% for autografts) with an economic benefit favoring allografts, especially in smaller nerve gaps [49]. Despite these promising results, direct comparisons between cable autografts and biological conduits are lacking, underscoring a critical gap in the existing literature [29]. This remains a significant gap, particularly in studies assessing motor outcomes in large nerve defects.

### 4.6. Limitations and Challenges

Despite promising motor outcomes, the current evidence base is limited by heterogeneity in study designs, inconsistent outcome measures, and the absence of randomized controlled trials. Registry data, such as those from the RANGER study, offer valuable insights but are inherently limited by selection bias and lack of standardization. While the presented clinical studies provide valuable insights, many are limited by retrospective design, small sample sizes, heterogeneous patient populations, and a lack of standardized motor outcome measures. Moreover, the risk of publication bias and the absence of randomized controlled trials reduce the strength of evidence, highlighting the need for prospective, high-quality studies with clearly defined endpoints and standardized follow-up protocols.

Despite substantial advancements, graft failures remain a significant concern. The heterogeneity of study populations—in terms of age, comorbidities, injury patterns, and rehabilitation protocols—makes it difficult to draw definitive conclusions or generate universal guidelines. Poor vascularization, infection, extensive trauma, and inappropriate patient selection have consistently been identified as major factors contributing to allograft failure [26,27,54].

Particularly challenging are cases involving extensive nerve damage, large gaps (>70 mm), or complex military blast injuries, where recovery rates are markedly lower [27]. This emphasizes the necessity for stringent patient selection criteria and tailored reconstruction approaches.

Moreover, most studies fail to report subgroup analyses for facial nerve outcomes, which further limits interpretability in this anatomically distinct context.

Future clinical studies should prioritize the facial nerve as a distinct target of investigation, with standardized functional assessments such as the House–Brackmann or Sunnybrook scale. Furthermore, translational strategies including stem cell augmentation, angiogenic scaffolds, or immunomodulatory approaches may help overcome the limitations observed in long-gap and complex reconstructions.

### 4.7. Research Advancements and Future Directions

Emerging research focuses on cellular and molecular strategies, such as stem cell augmentation, electrical stimulation, gene editing, and nanoengineered scaffolds, aiming to enhance nerve regeneration outcomes [41,42,43,44,45,53]. Promising results from preclinical studies suggest potential avenues for clinical translation, though significant hurdles remain concerning biocompatibility, efficacy, and ethical considerations.

Clinically, vascularized nerve scaffolds offer a promising direction to improve outcomes, especially in larger gaps where current options are limited [47]. The integration of these technologies in clinical practice remains speculative, pending rigorous validation through randomized controlled trials. Two early-phase clinical trials are currently investigating strategies for facial nerve reconstruction. One evaluates NTX-001 for the prevention of facial paralysis following surgery (NCT05293522), and the other examines intraoperative electrical stimulation during cross-facial nerve grafting (NCT06335719). These studies reflect a translational shift toward incorporating biologic and neuromodulatory techniques into clinical practice [56,57]. Collaborative, multicenter research is necessary to overcome the existing heterogeneity in patient populations and injury types, thereby advancing clinical evidence [8].

## 5. Conclusions

Processed nerve allografts have emerged as a clinically relevant alternative to autologous grafts for motor nerve repair, particularly in short to intermediate gap lengths. They offer key advantages, including the avoidance of donor-site morbidity and immediate availability. Clinical and experimental data suggest meaningful motor recovery in selected indications, with outcomes comparable to autografts in gaps less than 50–60 mm.

However, facial nerve reconstruction remains an underrepresented and poorly studied domain. Given the unique anatomical complexity and psychosocial significance of facial nerve function, extrapolation from upper extremity or mixed motor nerve data is insufficient. While preclinical studies support the feasibility of facial nerve regeneration using processed allografts, robust clinical trials are lacking. Future research should prioritize standardized outcome measures, dedicated subgroup analyses for facial nerve repairs, and translational strategies such as stem cell augmentation or vascularized scaffolds. Until then, the role of processed nerve allografts in facial nerve reconstruction should be approached with cautious optimism and rigorous clinical judgment.

## Figures and Tables

**Table 1:** Motor function recovery in the clinical setting of nerve allograft.

Reference, Year	N * Total	N ** Motor	Study Design	Nerve Type	Outcome (≥M3/M4)	Level of Evidence	Summary
Isaacs and Safa, 2017 [23]	15	15	Retrospective cohort	Motor	85%	III	Allografts of up to 5 mm in diameter appear capable of supporting successful nerve regeneration and recovery of motor function in 85%.
Cho et al., 2012 [22]	51	3	Registry analysis	Mixed	86%	III	Allografts are safe and effective, and meaningful recovery (S3/M4 or above) in 86%.
Safa et al., 2019 [16]	22	22	Registry analysis	Motor/Mixed	73%	III	Meaningful recovery of motor function of 73%, safe, and provided functional motor recovery in mixed and motor nerve repairs.
Safa et al., 2020 [17]	475	12	Registry analysis	Motor	82%	III	Support the use up to 70 mm, and meaningful recovery was achieved in 82%.
Brooks et al., 2012 [15]	76	9	Registry analysis	Mixed	Not specified	III	Allografts are safe and effective in sensory, mixed, and motor nerve defects (5–50 mm).
Carlson et al., 2018 [25]	16	5	Case series	Facial	33%	IV	Facial reanimation in two patients using cadaveric nerve allografts in babysitter procedure; both showed functional recovery of eye sphincter and lip depressors.
Squintani et al., 2013 [3]	14	14	Case series	Motor plexus	Not quantified	IV	Valid surgical strategy to restore function. New perspectives on procedures for extensive reconstruction of brachial and lumbosacral plexuses.
Zhu, 2017 [21]	64	6	Cohort study	Motor	66.7%	III	Meaningful recovery in 84.6% (sensory), 57.9% (mixed), and 66.7% (motor).

*: total number of nerves; **: number of motor nerves.

**Table 2:** Studies on facial nerve reconstruction using processed or acellular nerve allografts.

Author (Year)	Study Type	Animal/Human	Model/Indication	Intervention	Key Outcome	Notes
Hontanilla (2006) [5]	Preclinical	Primate	Facial nerve defect	Cold-preserved allograft + Tacrolimus	Functional recovery; 70% motoneuron loss after FK506 withdrawal	Clinical function preserved despite subclinical deficits
Hu (2010) [31]	Preclinical	Rabbit	Complete facial nerve defect	Processed facial nerve allograft	Partial recovery: technical feasibility demonstrated	Myelin thinning and disorganized architecture vs. autograft
Huang (2015) [39]	Preclinical	Rat	Facial nerve defect	Acellular xenograft vs. allograft	Both groups regenerated; xenograft slightly inferior	Lower axon density and myelin thickness
Carlson (2018) [25]	Clinical	Human	Facial nerve palsy (2 patients)	Cadaveric nerve allograft	Facial reanimation (2 patients) with cadaveric nerve allografts; recovery of eye sphincter and lip depressors	Very limited clinical data
Bengur (2022) [9]	Review	Preclinical	Facial nerve models	Bioengineered allografts	Promising in animal models	Scaffold design, stem cells, and growth factors discussed
Ali (2019) [34]	Preclinical	Rat	Facial nerve defect	Motor vs. sensory autografts	Motor autografts led to significantly better recovery	Highlights relevance of donor nerve identity
Zhu (2017) [21]	Preclinical	Primate	Complete facial nerve defect	Acellular xenograft (monkey)	Restoration of facial symmetry and muscle activation	Histological regeneration inferior to autograft
Leckenby (2020) [14]	Clinical Series	Human	Various peripheral nerve injuries	Avance^®^ allograft	Included facial nerve repairs; no subgroup data	Lacks facial nerve-specific outcome data
Peters (2023) [18]	Clinical Series	Human	Major nerve repairs (some cranial)	Acellular allograft	Recovery in select cases; overall variable	Facial nerves not specifically reported

**Table 3:** Indications, advantages, and disadvantages of allografts and autografts.

Category	Allograft	Autograft
Indications	Short to moderate nerve gaps (≤5 cm) where direct repair is not possible. Limited donor nerve availability due to prior surgeries or insufficient donor sites. Patients unsuitable for autografts (e.g., systemic conditions like diabetes and vascular disease). Multiple nerve injuries where autograft resources are insufficient. Preference for off-the-shelf options for quicker surgical intervention.	Critical or long nerve gaps (>5 cm) require robust conduits with live Schwann cells. High-function areas (motor nerves or optimal sensory recovery). Availability of healthy donor nerves (e.g., sural nerve, auricularis magnus nerve). Young or healthy patients with good healing potential and tolerance for donor-site morbidity. Complex defects require native tissue for better functional recovery.
Advantages	No donor-site morbidity (no additional surgical site). Readily available off-the-shelf options. Reduces surgery time.	Immunological compatibility (no risk of rejection). Gold standard with established efficacy (higher regeneration rates and functional recovery). Avoids limitations in allograft availability. Provides viable Schwann cells for neurotrophic support. Proven long-term success for motor and sensory nerves.
Disadvantages	Slower regeneration rates compared to autografts. Variability in graft quality and clinical outcomes. Limited data on long-term outcomes in motor nerve repairs.	Limited by the availability of donor nerves. Donor-site morbidity (e.g., sensory loss or scarring). Increased surgical complexity. Potential for complications at the donor site.
